# Supplementary material for: Association of Human Leukocyte Antigen Alleles with COVID-19 Severity and Mortality in a Spanish Population
Source: Medicina (Kaunas). 2024 Aug 25;60(9):1392. doi: 10.3390/medicina60091392 (PMC11434301; doi:10.3390/medicina60091392)
Supplement: Supplementary file 1 [file medicina-60-01392-s001.zip › Supplementary Table S1.pdf]

Supplementary Table 1. HLA allele frequencies between admitted and not admitted patients. P = p value (Fisher's exact test).

HLA-A

		**Total (n=190)**	**Admitted in hospital (n=169)**	**Not admitted in hospital (n=21)**	**P**
**1**	**A*01:01**	24/186 (12.9)	22/165 (13.3)	2/21 (9.5)	1.000
**2**	**A*01:02**	1/190 (0.5)	1/169 (0.6)	0/21 (0.0)	1.000
**3**	**A*02:01**	36/186 (19.4)	34/165 (20.6)	2/21 (9.5)	0.377
**4**	**A*02:02**	3/186 (1.6)	3/165 (1.8)	0/21 (0.0)	1.000
**5**	**A*02:05**	4/186 (2.2)	4/165 (2.4)	0/21 (0.0)	1.000
**6**	**A*03:01**	30/186 (16.1)	24/165 (14.5)	6/21 (28.6)	0.116
**7**	**A*03:02**	1/188 (0.5)	0/167 (0.0)	1/21 (4.8)	0.112
**8**	**A*11:01**	11/186 (5.9)	8/165 (4.8)	3/21 (14.3)	0.113
**9**	**A*23:01**	1/186 (0.5)	1/165 (0.6)	0/21 (0.0)	1.000
**10**	**A*24:02**	22/186 (11.8)	21/165 (12.7)	1/21 (4.8)	0.476
**11**	**A*25:01**	1/190 (0.5)	1/169 (0.6)	0/21 (0.0)	1.000
**12**	**A*26:01**	2/186 (1.1)	2/165 (1.2)	0/21 (0.0)	1.000
**13**	**A*29:02**	18/186 (9.7)	17/165 (10.3)	1/21 (4.8)	0.699
**14**	**A*30:01**	2/186 (1.1)	1/165 (0.6)	1/21 (4.8)	0.214
**15**	**A*30:02**	3/187 (1.6)	2/166 (1.2)	1/21 (4.8)	0.302
**16**	**A*31:01**	4/187 (2.1)	2/166 (1.2)	2/21 (9.5)	0.063
**17**	**A*32:01**	4/186 (2.2)	4/165 (2.4)	0/21 (0.0)	1.000
**18**	**A*33:01**	3/186 (1.6)	3/165 (1.8)	0/21 (0.0)	1.000
**19**	**A*33:03**	2/190 (1.1)	2/169 (1.2)	0/21 (0.0)	1.000
**20**	**A*66:01**	1/186 (0.5)	1/165 (0.6)	0/21 (0.0)	1.000
**21**	**A*68:01**	9/186 (4.8)	9/165 (5.5)	0/21 (0.0)	0.601
**22**	**A*68:02**	3/187 (1.6)	2/166 (1.2)	1/21 (4.8)	0.302

HLA-B

		Total (n=190)	Admitted in hospital (n=169)	Not admitted in hospital (n=21)	P
**1**	**B*07:02**	13/163 (8.0)	12/144 (8.3)	1/19 (5.3)	1.000
**2**	**B*07:05**	2/163 (1.2)	0/144 (0.0)	2/19 (10.5)	**0.013**
**3**	**B*08:01**	15/163 (9.2)	15/144 (10.4)	0/19 (0.0)	0.221
**4**	**B*13:02**	5/163 (3.1)	3/144 (2.1)	2/19 (10.5)	0.104
**5**	**B*14:01**	3/163 (1.8)	3/144 (2.1)	0/19 (0.0)	1.000
**6**	**B*14:02**	8/163 (4.9)	7/144 (4.9)	1/19 (5.3)	1.000
**7**	**B*15:01**	3/163 (1.8)	3/144 (2.1)	0/19 (0.0)	1.000
**8**	**B*15:04**	1/163 (0.6)	1/144 (0.7)	0/19 (0.0)	1.000
**9**	**B*15:17**	2/161 (1.2)	2/142 (1.4)	0/19 (0.0)	1.000
**10**	**B*18:01**	17/163 (10.4)	16/144 (11.1)	1/19 (5.3)	0.696
**11**	**B*27:05**	4/163 (2.5)	3/144 (2.1)	1/19 (5.3)	0.394
**12**	**B*35:01**	2/163 (1.2)	1/144 (0.7)	1/19 (5.3)	0.220
**13**	**B*35:03**	3/163 (1.8)	3/144 (2.1)	0/19 (0.0)	1.000
**14**	**B*35:43**	2/163 (1.2)	2/144 (1.4)	0/19 (0.0)	1.000
**15**	**B*37:01**	2/163 (1.2)	2/144 (1.4)	0/19 (0.0)	1.000
**16**	**B*38:01**	7/163 (4.3)	7/144 (4.8)	0/19 (0.0)	1.000
**17**	**B*39:06**	1/163 (0.6)	1/144 (0.7)	0/19 (0.0)	1.000
**18**	**B*40:01**	3/163 (1.8)	2/144 (1.4)	1/19 (5.3)	0.312
**19**	**B*40:02**	1/163 (0.6)	1/144 (0.7)	0/19 (0.0)	1.000
**20**	**B*40:06**	1/163 (0.6)	1/144 (0.7)	0/19 (0.0)	1.000
**21**	**B*41:01**	1/162 (0.6)	1/143 (0.7)	0/19 (0.0)	1.000
**22**	**B*41:02**	2/163 (1.2)	2/144 (1.4)	0/19 (0.0)	1.000
**23**	**B*42:01**	1/163 (0.6)	1/144 (0.7)	0/19 (0.0)	1.000
**24**	**B*44:02**	7/163 (4.3)	6/144 (4.2)	1/19 (5.3)	0.587
**25**	**B*44:03**	13/162 (8.0)	12/143 (8.4)	1/19 (5.3)	1.000
**26**	**B*45:01**	4/163 (2.5)	4/144 (2.8)	0/19 (0.0)	1.000
**27**	**B*46:01**	1/163 (0.6)	0/144 (0.0)	1/19 (5.3)	0.117

**28**	**B*47:01**	1/163 (0.6)	1/144 (0.7)	0/19 (0.0)	1.000
**29**	**B*48:01**	1/163 (0.6)	1/144 (0.7)	0/19 (0.0)	1.000
**30**	**B*49:01**	5/163 (3.1)	3/144 (2.1)	2/19 (10.5)	0.104
**31**	**B*50:01**	3/163 (1.8)	3/144 (2.1)	0/19 (0.0)	1.000
**32**	**B*51:01**	7/162 (4.9)	7/143 (4.8)	1/19 (5.3)	1.000
**33**	**B*52:01**	5/163 (3.1)	5/144 (3.5)	0/19 (0.0)	1.000
**34**	**B*53:01**	2/163 (1.2)	1/144 (0.7)	1/19 (5.3)	0.220
**35**	**B*55:01**	2/163 (1.2)	1/144 (0.7)	1/19 (5.3)	0.220
**36**	**B*57:01**	7/163 (4.3)	6/144 (4.2)	1/19 (5.3)	0.587
**37**	**B*58:01**	3/163 (1.8)	4/144 (2.8)	0/19 (0.0)	1.000

HLA-C

		Total (n=190)	Admitted in hospital (n=169)	Not admitted in hospital (n=21)	P
**1**	**C*01:02**	13/188 (6.9)	11/167 (6.6)	2/21 (9.5)	0.642
**2**	**C*02:02**	9/188 (4.8)	8/167 (4.8)	1/21 (4.8)	1.000
**3**	**C*03:02**	2/188 (1.1)	2/167 (1.2)	0/21 (0.0)	1.000
**4**	**C*03:03**	3/188 (1.6)	3/167 (1.8)	0/21 (0.0)	1.000
**5**	**C*03:04**	6/188 (3.7)	6/167 (3.6)	1/21 (4.8)	0.570
**6**	**C*04:01**	20/188 (10.6)	16/167 (9.6)	4/21 (19.0)	0.249
**7**	**C*05:01**	18/188 (9.6)	13/167 (7.8)	5/21 (23.8)	**0.035**
**8**	**C*06:02**	18/188 (9.6)	17/167 (10.2)	1/21 (4.8)	0.698
**9**	**C*07:01**	30/188 (16.0)	27/167 (16.2)	3/21 (14.3)	1.000
**10**	**C*07:02**	12/188 (6.4)	10/167 (6.0)	2/21 (9.5)	0.628
**11**	**C*07:04**	3/188 (1.6)	2/167 (1.2)	1/21 (4.8)	0.300
**12**	**C*08:01**	1/188 (0.5)	1/167 (0.6)	0/21 (0.0)	1.000
**13**	**C*08:02**	10/188 (5.3)	10/167 (6.0)	0/21 (0.0)	0.606
**14**	**C*12:02**	5/186 (2.7)	4/165 (2.4)	1/21 (4.8)	0.454
**15**	**C*12:03**	18/188 (9.6)	18/167 (10.8)	0/21 (0.0)	0.230
**16**	**C*14:02**	1/187 (0.5)	1/166 (0.6)	0/21 (0.0)	1.000
**17**	**C*15:02**	6/188 (3.2)	6/167 (3.6)	0/21 (0.0)	1.000
**18**	**C*16:01**	6/188 (3.2)	6/167 (3.6)	0/21 (0.0)	1.000
**19**	**C*16:02**	1/188 (0.5)	1/167 (0.6)	0/21 (0.0)	1.000
**20**	**C*17:01**	5/188 (2.7)	5/167 (3.0)	0/21 (0.0)	1.000

HLA-DPA1

		Total (n=190)	Admitted in hospital (n=169)	Not admitted in hospital (n=21)	P
**1**	**DPA1*01:03**	147/188 (78.2)	131/167 (78.4)	16/21 (76.2)	0.783
**2**	**DPA1*01:05**	1/188 (0.5)	1/167 (0.6)	0/21 (0.0)	1.000
**3**	**DPA1*02:01**	36/188 (19.1)	31/167 (18.6)	5/21 (23.8)	0.561
**4**	**DPA1*02:02**	3/188 (1.6)	3/167 (1.8)	0/21 (0.0)	1.000
**5**	**DPA1*03:01**	1/188 (0.5)	1/167 (0.6)	0/21 (0.0)	1.000

HLA-DPB1

		Total (n=190)	Admitted in hospital (n=169)	Not admitted in hospital (n=21)	P
1	DPB1*01:01	11/173 (6.9)	11/153 (7.2)	0/20 (0.0)	0.367
2	DPB1*02:01	26/173 (15.0)	24/153 (15.7)	2/20 (10.0)	0.742
3	DPB1*02:02	2/173 (1.2)	2/153 (1.3)	0/20 (0.0)	1.000
4	DPB1*03:01	10/173 (5.8)	8/153 (5.2)	2/20 (10.0)	0.325
5	DQB1*03:02	1/169 (0.6)	1/149 (0.7)	0/20 (0.0)	1.000
6	DPB1*04:01	58/173 (33.5)	49/153 (32.0)	9/20 (45.0)	0.314
7	DPB1*04:02	29/173 (16.7)	25/153 (16.3)	4/20 (20.0)	0.750
8	DPB1*05:01	3/173 (1.7)	3/153 (2.0)	0/20 (0.0)	1.000
9	DPB1*09:01	1/173 (0.6)	1/153 (0.7)	0/20 (0.0)	1.000
10	DPB1*10:01	3/173 (1.7)	3/153 (2.0)	0/20 (0.0)	1.000
11	DPB1*11:01	11/173 (6.9)	12/153 (7.8)	0/20 (0.0)	0.364
12	DPB1*13:01	5/173 (2.9)	5/153 (3.3)	0/20 (0.0)	1.000
13	DPB1*14:01	4/173 (2.3)	3/153 (2.0)	1/20 (5.0)	0.391
14	DPB1*15:01	1/173 (0.6)	1/153 (0.7)	0/20 (0.0)	1.000
15	DPB1*17:01	4/173 (2.3	3/153 (2.0)	2/20 (10.0)	0.103
16	DPB1*19:01	2/173 (1.2)	2/153 (1.3)	0/20 (0.0)	1.000

HLA-DQA1

		Total (n=190)	Admitted in hospital (n=169)	Not admitted in hospital (n=21)	P
**1**	**DQA1*01:01**	30/182 (16.5)	23/162 (14.2)	7/20 (35.0)	**0.027**
**2**	**DQA1*01:02**	35/182 (19.2)	32/162 (20.0)	3/20 (15.0)	0.769
**3**	**DQA1*01:03**	15/182 (8.2)	14/162 (8.6)	1/20 (5.0)	1.000
**4**	**DQA1*02:01**	28/182 (15.4)	22/162 (13.6)	6/20 (30.0)	0.092
**5**	**DQA1*03:01**	27/182 (14.8)	24/162 (14.8)	3/20 (15.0)	1.000
**6**	**DQA1*04:01**	6/182 (3.3)	6/162 (3.7)	0/20 (0.0)	1.000
**7**	**DQA1*05:01**	41/182 (22.5)	41/162 (25.3)	0/20 (0.0)	**0.008**

HLA-DQB1

		Total (n=190)	Admitted in hospital (n=169)	Not admitted in hospital (n=21)	P
**1**	**DQB1*02:01**	34/175 (19.4)	32/155 (20.6)	2/20 (10.0)	0.372
**2**	**DQB1*02:02**	3/175 (1.7)	1/155 (0.6)	2/20 (10.0)	**0.035**
**3**	**DQB1*03:01**	24/175 (13.7)	22/155 (14.2)	2/20 (10.0)	1.000
**4**	**DQB1*03:02**	18/175 (10.3)	18/155 (11.6)	0/20 (0.0)	0.231
**5**	**DQB1*03:03**	6/175 (3.4)	4/155 (2.6)	2/20 (10.0)	0.141
**6**	**DQB1*04:02**	9/175 (5.1)	9/155 (5.8)	0/20 (0.0)	0.600
**7**	**DQB1*05:01**	28/175 (16.0)	22/155 (14.2)	6/20 (30.0)	0.099
**8**	**DQB1*05:02**	5/175 (2.9)	5/155 (3.2)	0/20 (0.0)	1.000
**9**	**DQB1*05:03**	9/175 (5.1)	8/155 (5.2)	1/20 (5.0)	1.000
**10**	**DQB1*06:01**	4/175 (2.3)	4/155 (2.6)	0/20 (0.0)	1.000
**11**	**DQB1*06:02**	17/175 (9.7)	14/155 (9.0)	3/20 (15.0)	0.418
**12**	**DQB1*06:03**	9/175 (5.1)	8/155 (5.2)	1/20 (5.0)	1.000
**13**	**DQB1*06:04**	8/175 (4.6)	7/155 (4.5)	1/20 (5.0)	1.000
**14**	**DQB1*06:09**	1/175 (0.6)	1/155 (0.6)	0/20 (0.0)	1.000

HLA-DRB1

		Total (n=190)	Admitted in hospital (n=169)	Not admitted in hospital (n=21)	P
1	**DRB1*01:01**	6/160 (3.8)	5/142 (3.5)	1/18 (5.6)	0.517
2	**DRB1*01:02**	6/160 (3.8)	6/142 (4.2)	0/18 (0.0)	1.000
3	**DRB1*01:03**	2/160 (1.3)	1/142 (0.7)	1/18 (5.6)	0.213
4	**DRB1*03:01**	28/160 (17.5)	27/142 (19.0)	1/18 (5.6)	0.203
5	**DRB1*03:02**	1/160 (0.6)	1/142 (0.7)	0/18 (0.0)	1.000
6	**DRB1*04:01**	1/159 (0.6)	1/141 (0.7)	0/18 (0.0)	1.000
7	**DRB1*04:02**	2/160 (1.3)	2/142 (1.4)	0/18 (0.0)	1.000
8	**DRB1*04:04**	2/160 (1.3)	2/142 (1.4)	0/18 (0.0)	1.000
9	**DRB1*04:05**	1/160 (0.6)	0/142 (0.0)	1/18 (5.6)	0.112
10	**DRB1*04:07,**	0/159 (0.0)	0/141 (0.0)	0/18 (0.0)	1.000
11	**DRB1*04:10**	1/159 (0.6)	1/141 (0.7)	0/18 (0.0)	1.000
12	**DRB1*07:01**	32/160 (20.0)	25/142 (17.6)	7/18 (38.9)	0.055
13	**DRB1*08:01**	2/160 (1.3)	2/142 (1.4)	0/18 (0.0)	1.000
14	**DRB1*08:02**	1/159 (0.6)	1/141 (0.7)	0/18 (0.0)	1.000
15	**DRB1*08:04**	1/159 (0.6)	1/141 (0.7)	0/18 (0.0)	1.000
16	**DRB1*09:01**	2/160 (1.3)	1/142 (0.7)	1/18 (5.6)	0.213
17	**DRB1*10:01**	5/160 (3.1)	4/142 (2.8)	1/18 (5.6)	0.454
18	**DRB1*11:01**	3/160 (1.9)	2/142 (1.4)	1/18 (5.6)	0.303
19	**DRB1*11:02**	2/160 (1.3)	2/142 (1.4)	0/18 (0.0)	1.000
20	**DRB1*11:04**	4/160 (2.5)	4/142 (2.8)	0/18 (0.0)	1.000
21	**DRB1*11:06**	1/160 (0.6)	1/142 (0.7)	0/18 (0.0)	1.000
22	**DRB1*12:01**	1/159 (0.6)	1/141 (0.7)	0/18 (0.0)	1.000
23	**DRB1*13:01**	15/160 (9.4)	13/142 (9.2)	2/18 (11.1)	0.678
24	**DRB1*13:02**	3/160 (1.9)	3/142 (2.1)	0/18 (0.0)	1.000
25	**DRB1*14:01**	2/160 (1.3)	1/142 (0.7)	1/18 (5.6)	0.213
26	**DRB1*14:02**	4/160 (2.5)	4/142 (2.8)	0/18 (0.0)	1.000
27	**DRB1*14:04**	4/159 (2.5)	4/141 (2.8)	0/18 (0.0)	1.000

**28**	**DRB1*15:01**	13/160 (8.1)	12/142 (8.5)	1/18 (5.6)	1.000
**29**	**DRB1*15:02**	4/160 (2.5)	4/142 (2.8)	0/18 (0.0)	1.000
**30**	**DRB1*15:03**	1/159 (0.6)	1/141 (0.7)	0/18 (0.0)	1.000
**31**	**DRB1*16:01**	6/159 (3.8)	6/141 (4.3)	0/18 (0.0)	1.000
**32**	**DRB1*16:02**	1/159 (0.6)	1/141 (0.7)	0/18 (0.0)	1.000
**33**	**DRB3*99:01**	1/159 (0.6)	1/141 (0.7)	0/18 (0.0)	1.000

HLA-DRB3

		Total (n=190)	Admitted in hospital (n=169)	Not admitted in hospital (n=21)	P
1	**DRB3*01:01**	22/183 (12.0)	21/163 (12.9)	1/20 (5.0)	0.475
2	**DRB3*02:02**	43/183 (23.5)	38/163 (23.3)	5/20 (25.0)	0.788
3	**DRB3*03:01**	8/183 (4.4)	7/163 (4.3)	1/20 (5.0)	1.000
4	**DRB3*99:01**	110/183 (60.1)	97/163 (59.5)	13/20 (65.0)	0.810

HLA-DRB4

		Total (n=190)	Admitted in hospital (n=169)	Not admitted in hospital (n=21)	P
**1**	**DRB4*01:01**	5/183 (2.7)	5/163 (3.1)	0/20 (0.0)	1.000
**2**	**DRB4*01:03**	20/183 (10.9)	15/163 (9.2)	5/20 (25.0)	**0.049**
**3**	**DRB4*99:01**	158/183 (86.7)	143/163 (87.7)	15/20 (75.0)	0.151

HLA-DRB5

		Total (n=190)	Admitted in hospital (n=169)	Not admitted in hospital (n=21)	P
**1**	**DRB5*01:01**	19/188 (10.1)	16/167 (9.6)	3/21 (14.3)	0.451
**2**	**DRB5*01:02**	3/188 (1.6)	3/167 (1.8)	0/21 (0.0)	1.000
**3**	**DRB5*99:01**	166/188 (88.3)	148/167 (88.6)	18/21 (85.7)	0.718
