# Supplementary material for: Association of Human Leukocyte Antigen Alleles with COVID-19 Severity and Mortality in a Spanish Population
Source: Medicina (Kaunas). 2024 Aug 25;60(9):1392. doi: 10.3390/medicina60091392 (PMC11434301; doi:10.3390/medicina60091392)
Supplement: Supplementary file 1 [file medicina-60-01392-s001.zip › Supplementary Table S2.pdf]

Supplementary Table 2. HLA allele frequencies between patients with ICU admission or death (Yes ICU/D) and without ICU admission or death (No ICU/D). P = p value (Fisher's exact test).

HLA-A

		**Total (n=190)**	**Yes ICU/D (n=59)**	**No ICU/D (n=131)**	**P**
**1**	**A*01:01**	24/186 (12.9)	7/57 (12.3)	17/129 (13.2)	1.000
**2**	**A*01:02**	1/188 (0.5)	0/59 (0.0)	1/129 (0.8)	1.000
**3**	**A*02:01**	36/186 (19.4)	13/57 (22.8)	23/129 (17.2)	0.428
**4**	**A*02:02**	3/186 (1.6)	1/57 (1.8)	2/129 (1.6)	1.000
**5**	**A*02:05**	4/186 (2.2)	2/57 (3.5)	2/129 (1.6)	0.587
**6**	**A*03:01**	30/186 (16.1)	5/57 (8.8)	25/129 (19.4)	0.085
**7**	**A*03:02**	1/188 (0.5)	0/57 (0.0)	1/129 (0.8)	1.000
**8**	**A*11:01**	11/186 (5.9)	3/57 (5.3)	8/129 (6.2)	1.000
**9**	**A*23:01**	1/186 (0.5)	1/57 (1.8)	0/129 (0.0)	0.306
**10**	**A*24:02**	22/186 (11.8)	10/57 (17.5)	12/129 (9.3)	0.139
**11**	**A*25:01**	1/186 (0.5)	0/57 (0.0)	1/129 (0.8)	1.000
**12**	**A*26:01**	2/186 (1.1)	1/57 (1.8)	1/129 (0.8)	0.520
**13**	**A*29:02**	18/186 (9.7)	6/57 (10.5)	12/129 (9.3)	0.792
**14**	**A*30:01**	2/186 (1.1)	0/57 (0.0)	2/129 (1.6)	1.000
**15**	**A*30:02**	3/187 (1.6)	0/58 (0.0)	3/129 (2.3)	0.553
**16**	**A*31:01**	4/187 (2.1)	0/58 (0.0)	4/129 (3.1)	0.312
**17**	**A*32:01**	4/186 (2.2)	2/57 (3.5)	2/129 (1.6)	0.587
**18**	**A*33:01**	3/186 (1.6)	2/57 (3.5)	1/129 (0.8)	0.223
**19**	**A*33:03**	2/186 (1.1)	0/57 (0.0)	2/129 (1.6)	1.000
**20**	**A*66:01**	1/186 (0.5)	1/57 (1.8)	0/129 (0.0)	0.306
**21**	**A*68:01**	9/186 (4.8)	2/57 (3.5)	7/129 (5.4)	0.724
**22**	**A*68:02**	3/187 (1.6)	0/57 (0.0)	3/129 (2.3)	0.553

HLA-B

		Total (n=190)	Yes ICU/D (n=59)	No ICU/D (n=131)	P
**1**	**B*07:02**	13/163 (8.0)	4/50 (8.0)	9/113 (8.0)	1.000
**2**	**B*07:05**	2/163 (1.2)	0/50 (0.0)	2/113 (1.8)	1.000
**3**	**B*08:01**	15/163 (9.2)	2/50 (4.0)	13/113 (11.5)	0.152
**4**	**B*13:02**	5/163 (3.1)	0/50 (0.0)	5/113 (4.4)	0.325
**5**	**B*14:01**	3/163 (1.8)	3/50 (6.0)	0/113 (0.0)	**0.028**
**6**	**B*14:02**	8/163 (4.9)	0/50 (0.0)	8/113 (7.1)	0.108
**7**	**B*15:01**	3/163 (1.8)	1/50 (2.0)	2/113 (1.8)	1.000
**8**	**B*15:04**	1/163 (0.6)	0/50 (0.0)	1/113 (0.9)	1.000
**9**	**B*15:17**	2/161 (1.2)	2/47 (4.1)	0/112 (0.0)	0.091
**10**	**B*18:01**	17/163 (10.4)	7/50 (14.0)	10/113 (8.8)	0.405
**11**	**B*27:05**	4/163 (2.5)	1/50 (2.0)	3/113 (2.7)	1.000
**12**	**B*35:01**	2/163 (1.2)	0/50 (0.0)	2/113 (1.8)	1.000
**13**	**B*35:03**	3/163 (1.8)	3/50 (6.0)	0/112 (0.0)	**0.028**
**14**	**B*35:43**	2/163 (1.2)	0/50 (0.0)	2/113 (1.8)	1.000
**15**	**B*37:01**	2/163 (1.2)	1/49 (2.0)	1/113 (0.9)	0.515
**16**	**B*38:01**	7/163 (4.3)	3/50 (6.0)	4/113 (3.5)	0.440
**17**	**B*39:06**	1/163 (0.6)	0/50 (0.0)	1/113 (0.9)	1.000
**18**	**B*40:01**	3/163 (1.8)	1/50 (2.0)	2/113 (1.8)	1.000
**19**	**B*40:02**	1/163 (0.6)	1/50 (2.0)	0/112 (0.0)	0.307
**20**	**B*40:06**	1/163 (0.6)	0/50 (0.0)	1/113 (0.9)	1.000
**21**	**B*41:01**	1/162 (0.6)	1/49 (2.0)	0/112 (0.0)	0.302
**22**	**B*41:02**	2/163 (1.2)	0/50 (0.0)	2/113 (1.8)	1.000
**23**	**B*42:01**	1/163 (0.6)	1/50 (2.0)	0/112 (0.0)	0.307
**24**	**B*44:02**	7/163 (4.3)	3/50 (6.0)	4/113 (3.5)	0.440
**25**	**B*44:03**	13/162 (8.0)	5/49 (10.2)	8/113 (7.1)	0.535
**26**	**B*45:01**	4/163 (2.5)	0/50 (0.0)	4/113 (3.5)	0.313
**27**	**B*46:01**	1/163 (0.6)	0/50 (0.0)	1/112 (0.9)	1.000
**28**	**B*47:01**	1/163 (0.6)	1/50 (2.0)	0/112 (0.0)	0.307

**29**	**B*48:01**	1/163 (0.6)	0/50 (0.0)	1/113 (0.9)	1.000
**30**	**B*49:01**	5/163 (3.1)	0/50 (0.0)	5/113 (4.4)	0.325
**31**	**B*50:01**	3/163 (1.8)	0/50 (0.0)	3/113 (2.7)	0.553
**32**	**B*51:01**	7/162 (4.9)	2/49 (4.1)	6/113 (5.3)	1.000
**33**	**B*52:01**	5/163 (3.1)	2/50 (4.0)	3/113 (2.7)	0.643
**34**	**B*53:01**	2/163 (1.2)	0/50 (0.0)	2/113 (1.8)	1.000
**35**	**B*55:01**	2/163 (1.2)	1/50 (2.0)	1/112 (0.9)	0.521
**36**	**B*57:01**	7/163 (4.3)	2/50 (4.0)	5/113 (4.4)	1.000
**37**	**B*58:01**	3/163 (1.8)	2/50 (4.0)	2/113 (1.8)	0.587

HLA-C

		Total (n=190)	Yes ICU/D (n=59)	No ICU/D (n=131)	P
**1**	**C*01:02**	13/188 (6.9)	4/58 (6.9)	9/130 (6.9)	1.000
**2**	**C*02:02**	9/188 (4.8)	3/58 (5.2)	6/130 (4.6)	1.000
**3**	**C*03:02**	2/188 (1.1)	0/58 (0.0)	2/130 (1.5)	1.000
**4**	**C*03:03**	3/188 (1.6)	2/58 (3.4)	1/130 (0.8)	0.225
**5**	**C*03:04**	6/188 (3.7)	1/58 (1.7)	6/130 (4.6)	0.440
**6**	**C*04:01**	20/188 (10.6)	6/58 (10.3)	14/130 (10.8)	1.000
**7**	**C*05:01**	18/188 (9.6)	5/58 (8.6)	13/130 (10.0)	1.000
**8**	**C*06:02**	18/188 (9.6)	5/58 (8.6)	13/130 (10.0)	1.000
**9**	**C*07:01**	30/188 (16.0)	10/58 (17.2)	20/130 (15.4)	0.830
**10**	**C*07:02**	12/188 (6.4)	2/58 (3.4)	10/130 (7.7)	0.349
**11**	**C*07:04**	3/188 (1.6)	2/58 (3.4)	1/130 (0.8)	0.225
**12**	**C*08:01**	1/188 (0.5)	0/58 (0.0)	1/130 (0.8)	1.000
**13**	**C*08:02**	10/188 (5.3)	3/58 (5.2)	7/130 (5.4)	1.000
**14**	**C*12:02**	5/186 (2.7)	1/56 (1.8)	4/130 (3.1)	1.000
**15**	**C*12:03**	18/188 (9.6)	7/58 (12.1)	11/130 (8.5)	0.432
**16**	**C*14:02**	1/187 (0.5)	0/57 (0.0)	1/130 (0.8)	1.000
**17**	**C*15:02**	6/188 (3.2)	2/58 (3.4)	4/130 (3.1)	1.000
**18**	**C*16:01**	6/188 (3.2)	2/58 (3.4)	4/130 (3.1)	1.000
**19**	**C*16:02**	1/188 (0.5)	1/58 (1.7)	0/130 (0.0)	0.309
**20**	**C*17:01**	5/188 (2.7)	2/58 (3.4)	3/130 (2.3)	0.645

HLA-DPA1

		Total (n=190)	Yes ICU/D (n=59)	No ICU/D (n=131)	P
**1**	**DPA1*01:03**	147/188 (78.2)	48/59 (81.4)	99/129 (76.7)	0.570
**2**	**DPA1*01:05**	1/188 (0.5)	1/59 (1.7)	0/129 (0.0)	0.314
**3**	**DPA1*02:01**	36/188 (19.1)	7/59 (11.9)	29/129 (22.5)	0.110
**4**	**DPA1*02:02**	3/188 (1.6)	3/59 (5.1)	0/129 (0.0)	**0.030**
**5**	**DPA1*03:01**	1/188 (0.5)	0/59 (0.0)	1/129 (0.8)	1.000

HLADPB1

		Total (n=190)	Yes ICU/D (n=59)	No ICU/D (n=131)	P
**1**	**DPB1*01:01**	11/173 (6.9)	3/54 (5.6)	8/119 (6.7)	1.000
**2**	**DPB1*02:01**	26/173 (15.0)	12/54 (22.2)	14/119 (11.8)	0.106
**3**	**DPB1*02:02**	2/173 (1.2)	0/54 (0.0)	2/119 (1.7)	1.000
**4**	**DPB1*03:01**	10/173 (5.8)	1/54 (1.9)	9/119 (7.6)	0.175
**5**	**DPB1*03:02**	1/169 (0.6)	0/54 (0.0)	1/115 (0.9)	1.000
**6**	**DPB1*04:01**	58/173 (33.5)	18/54 (33.3)	40/119 (33.6)	1.000
**7**	**DPB1*04:02**	29/173 (16.7)	7/54 (13.0)	22/119 (18.5)	0.510
**8**	**DPB1*05:01**	3/173 (1.7)	2/54 (3.7)	1/119 (0.8)	0.230
**9**	**DPB1*09:01**	1/173 (0.6)	0/54 (0.0)	1/119 (0.8)	1.000
**10**	**DPB1*10:01**	3/173 (1.7)	1/54 (1.9)	2/119 (1.7)	1.000
**11**	**DPB1*11:01**	11/173 (6.9)	3/54 (5.6)	10/119 (7.6)	0.756
**12**	**DPB1*13:01**	5/173 (2.9)	2/54 (3.7)	3/119 (2.5)	0.648
**13**	**DPB1*14:01**	4/173 (2.3)	2/54 (3.7)	2/119 (1.7)	0.590
**14**	**DPB1*15:01**	1/173 (0.6)	1/54 (1.9)	0/119 (0.0)	0.312
**15**	**DPB1*17:01**	4/173 (2.3	0/54 (0.0)	5/119 (4.2)	0.326
**16**	**DPB1*19:01**	2/173 (1.2)	1/54 (1.9)	1/119 (0.8)	0.528

HLA-DQA1

		Total (n=190)	Yes ICU/D (n=59)	No ICU/D (n=131)	P
1	**DQA1*01:01**	30/182 (16.5)	8/54 (14.8)	22/128 (17.2)	0.828
2	**DQA1*01:02**	35/182 (19.2)	7/54 (13.0)	28/128 (21.9)	0.217
3	**DQA1*01:03**	15/182 (8.2)	4/54 (7.4)	11/128 (8.6)	1.000
4	**DQA1*02:01**	28/182 (15.4)	5/54 (9.3)	23/128 (18.0)	0.178
5	**DQA1*03:01**	27/182 (14.8)	11/54 (20.4)	16/128 (12.5)	0.178
6	**DQA1*04:01**	6/182 (3.3)	0/54 (0.0)	6/128 (4.7)	0.181
7	**DQA1*05:01**	41/182 (22.5)	19/54 (35.2)	22/128 (17.2)	**0.011**

HLA-DQB1

		Total (n=190)	Yes ICU/D (n=59)	No ICU/D (n=131)	P
1	DQB1*02:01	34/175 (19.4)	13/53 (24.5)	21/122 (17.2)	0.300
2	DQB1*02:02	3/175 (1.7)	0/53 (0.0)	3/122 (2.5)	0.554
3	DQB1*03:01	24/175 (13.7)	9/53 (17.0)	15/122 (12.3)	0.474
4	DQB1*03:02	18/175 (10.3)	8/53 (15.1)	10/122 (8.2)	0.182
5	DQB1*03:03	6/175 (3.4)	1/53 (1.9)	5/122 (4.1)	0.669
6	DQB1*04:02	9/175 (5.1)	1/53 (1.9)	8/122 (6.6)	0.280
7	DQB1*05:01	28/175 (16.0)	5/53 (9.4)	23/122 (18.9)	0.177
8	DQB1*05:02	5/175 (2.9)	1/53 (1.9)	4/122 (3.3)	1.000
9	DQB1*05:03	9/175 (5.1)	5/53 (9.4)	4/122 (3.3)	0.132
10	DQB1*06:01	4/175 (2.3)	0/53 (0.0)	4/122 (3.3)	0.316
11	DQB1*06:02	17/175 (9.7)	6/53 (11.3)	11/122 (9.0)	0.782
12	DQB1*06:03	9/175 (5.1)	2/53 (3.8)	7/122 (5.7)	0.725
13	DQB1*06:04	8/175 (4.6)	2/53 (3.8)	6/122 (4.9)	1.000
14	DQB1*06:09	1/175 (0.6)	0/53 (0.0)	1/122 (0.8)	1.000

HLA-DRB1

		Total (n=190)	Yes ICU/D (n=59)	No ICU/D (n=131)	P
**1**	**DRB1*01:01**	6/160 (3.8)	3/48 (6.3)	3/112 (2.7)	0.366
**2**	**DRB1*01:02**	6/160 (3.8)	3/48 (6.3)	3/112 (2.7)	0.366
**3**	**DRB1*01:03**	2/160 (1.3)	0/48 (0.0)	2/112 (1.8)	1.000
**4**	**DRB1*03:01**	28/160 (17.5)	12/48 (25.0)	16/112 (14.3)	0.115
**5**	**DRB1*03:02**	1/160 (0.6)	1/48 (2.1)	0/112 (0.0)	0.300
**6**	**DRB1*04:01**	1/159 (0.6)	0/48 (0.0)	1/111 (0.9)	1.000
**7**	**DRB1*04:02**	2/160 (1.3)	2/48 (4.2)	0/112 (0.0)	0.089
**8**	**DRB1*04:04**	2/160 (1.3)	2/48 (4.2)	0/112 (0.0)	0.089
**9**	**DRB1*04:05**	1/160 (0.6)	0/48 (0.0)	1/112 (0.9)	1.000
**10**	**DRB1*04:07,**	0/159 (0.0)	0/48 (0.0)	0/111 (0.0)	1.000
**11**	**DRB1*04:10**	1/159 (0.6)	0/48 (0.0)	1/111 (0.9)	1.000
**12**	**DRB1*07:01**	32/160 (20.0)	7/48 (14.6)	25/112 (22.3)	0.290
**13**	**DRB1*08:01**	2/160 (1.3)	0/48 (0.0)	2/112 (1.8)	1.000
**14**	**DRB1*08:02**	1/159 (0.6)	0/48 (0.0)	1/111 (0.9)	1.000
**15**	**DRB1*08:04**	1/159 (0.6)	0/48 (0.0)	1/111 (0.9)	1.000
**16**	**DRB1*09:01**	2/160 (1.3)	0/48 (0.0)	2/112 (1.8)	1.000
**17**	**DRB1*10:01**	5/160 (3.1)	0/48 (0.0)	5/112 (4.5)	0.323
**18**	**DRB1*11:01**	3/160 (1.9)	0/48 (0.0)	3/112 (2.7)	0.555
**19**	**DRB1*11:02**	2/160 (1.3)	1/48 (2.1)	1/112 (0.9)	0.511
**20**	**DRB1*11:04**	4/160 (2.5)	2/48 (4.2)	2/112 (1.8)	0.584
**21**	**DRB1*11:06**	1/160 (0.6)	3/48 (6.3)	0/112 (0.0)	0.300
**22**	**DRB1*12:01**	1/159 (0.6)	0/48 (0.0)	1/111 (0.9)	1.000
**23**	**DRB1*13:01**	15/160 (9.4)	2/48 (4.2)	12/112 (10.7)	0.556
**24**	**DRB1*13:02**	3/160 (1.9)	1/48 (2.1)	2/112 (1.8)	1.000
**25**	**DRB1*14:01**	2/160 (1.3)	0/48 (0.0)	2/112 (1.8)	1.000
**26**	**DRB1*14:02**	4/160 (2.5)	0/48 (0.0)	4/112 (3.6)	0.317
**27**	**DRB1*14:04**	4/159 (2.5)	1/48 (2.1)	3/111 (2.7)	1.000
**28**	**DRB1*15:01**	13/160 (8.1)	8/48 (16.7)	5/112 (4.5)	**0.022**

**29**	**DRB1*15:02**	4/160 (2.5)	1/48 (2.1)	3/112 (2.7)	1.000
**30**	**DRB1*15:03**	1/159 (0.6)	0/48 (0.0)	1/111 (0.9)	1.000
**31**	**DRB1*16:01**	6/159 (3.8)	1/48 (2.1)	5/111 (4.5)	0.669
**32**	**DRB1*16:02**	1/159 (0.6)	0/48 (0.0)	1/111 (0.9)	1.000
**33**	**DRB3*99:01**	1/159 (0.6)	0/48 (0.0)	1/111 (0.9)	1.000

HLA-DRB3

		Total (n=190)	Yes ICU/D (n=59)	No ICU/D (n=131)	P
1	DRB3*01:01	22/183 (12.0)	10/59 (17.0)	12/124 (9.7)	0.223
2	DRB3*02:02	43/183 (23.5)	14/59 (23.7)	29/124 (23.4)	1.000
3	DRB3*03:01	8/183 (4.4)	3/59 (5.1)	5/124 (4.0)	0.714
4	DRB3*99:01	110/183 (60.1)	32/59 (54.2)	78/124 (62.9)	0.333

HLA-DRB4

		Total (n=190)	Yes ICU/D (n=59)	No ICU/D (n=131)	P
**1**	**DRB4*01:01**	5/183 (2.7)	3/58 (5.2)	2/125 (1.6)	0.328
**2**	**DRB4*01:03**	20/183 (10.9)	1/58 (1.7)	19/125 (15.2)	**0.005**
**3**	**DRB4*99:01**	158/183 (86.7)	54/58 (93.1)	104/125 (83.9)	0.103

HLA-DRB5

		Total (n=190)	Yes ICU/D (n=59)	No ICU/D (n=131)	P
**1**	**DRB5*01:01**	19/188 (10.1)	6/58 (10.3)	13/130 (10.0)	1.000
**2**	**DRB5*01:02**	3/188 (1.6)	1/58 (1.7)	2/130 (1.5)	1.000
**3**	**DRB5*99:01**	166/188 (88.3)	51/58 (87.9)	115/130 (88.5)	1.000
