# Supplementary material for: Association of Human Leukocyte Antigen Alleles with COVID-19 Severity and Mortality in a Spanish Population
Source: Medicina (Kaunas). 2024 Aug 25;60(9):1392. doi: 10.3390/medicina60091392 (PMC11434301; doi:10.3390/medicina60091392)
Supplement: Supplementary file 1 [file medicina-60-01392-s001.zip › Supplementary Table S3.pdf]

Supplementary Table 3. HLA allele frequencies between patients who died and those who survived. P = p value (Fisher's exact test).

HLA-A

		**Total (n=190)**	**Died (n=30)**	**Survived (n=160)**	**P**
**1**	**A*01:01**	24/186 (12.9)	4/30 (13.3)	20/156 (12.8)	1.000
**2**	**A*01:02**	1/190 (0.5)	0/30 (0.0)	1/160 (0.6)	1.000
**3**	**A*02:01**	36/186 (19.4)	8/30 (26.7)	28/156 (17.9)	0.313
**4**	**A*02:02**	3/186 (1.6)	0/30 (0.0)	3/156 (1.9)	1.000
**5**	**A*02:05**	4/186 (2.2)	1/30 (3.3)	3/156 (1.9)	0.508
**6**	**A*03:01**	30/186 (16.1)	2/30 (6.7)	28/156 (17.9)	0.176
**7**	**A*03:02**	1/188 (0.5)	0/30 (0.0)	1/158 (0.6)	1.000
**8**	**A*11:01**	11/186 (5.9)	2/30 (6.7)	9/156 (5.8)	0.692
**9**	**A*23:01**	1/186 (0.5)	0/30 (0.0)	1/156 (0.6)	1.000
**10**	**A*24:02**	22/186 (11.8)	5/30 (16.7)	17/156 (10.9)	0.362
**11**	**A*25:01**	1/190 (0.5)	0/30 (0.0)	1/160 (0.6)	1.000
**12**	**A*26:01**	2/186 (1.1)	1/30 (3.3)	1/156 (0.6)	0.297
**13**	**A*29:02**	18/186 (9.7)	4/30 (13.3)	14/156 (9.0)	0.499
**14**	**A*30:01**	2/186 (1.1)	0/30 (0.0)	2/156 (1.3)	1.000
**15**	**A*30:02**	3/187 (1.6)	0/30 (0.0)	3/157 (1.9)	1.000
**16**	**A*31:01**	4/187 (2.1)	0/30 (0.0)	4/157 (2.5)	1.000
**17**	**A*32:01**	4/186 (2.2)	0/30 (0.0)	4/156 (2.6)	1.000
**18**	**A*33:01**	3/186 (1.6)	2/30 (6.7)	1/156 (0.6)	0.068
**19**	**A*33:03**	2/190 (1.1)	0/30 (0.0)	2/160 (1.3)	1.000
**20**	**A*66:01**	1/186 (0.5)	0/30 (0.0)	1/156 (0.6)	1.000
**21**	**A*68:01**	9/186 (4.8)	0/30 (0.0)	9/156 (5.8)	0.359
**22**	**A*68:02**	3/187 (1.6)	0/30 (0.0)	3/157 (1.9)	1.000

HLA-B

		Total (n=190)	Died (n=30)	Survived (n=160)	P
1	B*07:02	13/163 (8.0)	1/26 (3.8)	12/137 (8.8)	0.695
2	B*07:05	2/163 (1.2)	0/26 (0.0)	2/137 (1.5)	1.000
3	B*08:01	15/163 (9.2)	2/26 (7.7)	13/137 (9.5)	1.000
4	B*13:02	5/163 (3.1)	0/26 (0.0)	5/137 (3.6)	1.000
5	B*14:01	3/163 (1.8)	2/26 (7.7)	1/137 (0.7)	0.067
6	B*14:02	8/163 (4.9)	0/26 (0.0)	8/137 (5.8)	0.357
7	B*15:01	3/163 (1.8)	0/26 (0.0)	3/137 (2.2)	1.000
8	B*15:04	1/163 (0.6)	0/26 (0.0)	1/137 (0.7)	1.000
9	B*15:17	2/161 (1.2)	1/26 (3.8)	1/135 (0.7)	0.298
10	B*18:01	17/163 (10.4)	2/26 (7.7)	15/137 (10.9)	1.000
11	B*27:05	4/163 (2.5)	0/26 (0.0)	4/137 (2.9)	1.000
12	B*35:01	2/163 (1.2)	0/26 (0.0)	2/137 (1.5)	1.000
13	B*35:03	3/163 (1.8)	3/26 (11.5)	0/137 (0.0)	**0.004**
14	B*35:43	2/163 (1.2)	0/26 (0.0)	2/137 (1.5)	1.000
15	B*37:01	2/163 (1.2)	0/25 (0.0)	2/138 (1.4)	1.000
16	B*38:01	7/163 (4.3)	2/26 (7.7)	5/137 (3.6)	0.310
17	B*39:06	1/163 (0.6)	0/26 (0.0)	1/137 (0.7)	1.000
18	B*40:01	3/163 (1.8)	1/26 (3.8)	2/137 (1.5)	0.408
19	B*40:02	1/163 (0.6)	0/26 (0.0)	1/137 (0.7)	1.000
20	B*40:06	1/163 (0.6)	0/26 (0.0)	1/137 (0.7)	1.000
21	B*41:01	1/162 (0.6)	0/25 (0.0)	1/137 (0.7)	1.000
22	B*41:02	2/163 (1.2)	0/26 (0.0)	2/137 (1.5)	1.000
23	B*42:01	1/163 (0.6)	1/26 (3.8)	0/137 (0.0)	0.160
24	B*44:02	7/163 (4.3)	2/26 (7.7)	5/137 (3.6)	0.310
25	B*44:03	13/162 (8.0)	4/25 (16.0)	9/137 (6.6)	0.119
26	B*45:01	4/163 (2.5)	0/26 (0.0)	4/137 (2.9)	1.000
27	B*46:01	1/163 (0.6)	0/26 (0.0)	1/137 (0.7)	1.000
28	B*47:01	1/163 (0.6)	1/26 (3.8)	0/137 (0.0)	0.160
29	B*48:01	1/163 (0.6)	0/26 (0.0)	1/137 (0.7)	1.000

**30**	**B*49:01**	5/163 (3.1)	0/26 (0.0)	5/137 (3.6)	1.000
**31**	**B*50:01**	3/163 (1.8)	0/26 (0.0)	3/137 (2.2)	1.000
**32**	**B*51:01**	7/162 (4.9)	0/25 (0.0)	8/137 (5.8)	0.610
**33**	**B*52:01**	5/163 (3.1)	0/26 (0.0)	5/137 (3.6)	1.000
**34**	**B*53:01**	2/163 (1.2)	0/26 (0.0)	2/137 (1.5)	1.000
**35**	**B*55:01**	2/163 (1.2)	1/26 (3.8)	1/137 (0.7)	0.294
**36**	**B*57:01**	7/163 (4.3)	1/26 (3.8)	6/137 (4.4)	1.000
**37**	**B*58:01**	3/163 (1.8)	2/26 (7.7)	2/137 (1.5)	0.120

HLA-C

		Total (n=190)	Died (n=30)	Survived (n=160)	P
**1**	**C*01:02**	13/188 (6.9)	3/30 (10.0)	10/158 (6.3)	0.440
**2**	**C*02:02**	9/188 (4.8)	1/30 (3.3)	8/158 (5.1)	1.000
**3**	**C*03:02**	2/188 (1.1)	0/30 (0.0)	2/158 (1.3)	1.000
**4**	**C*03:03**	3/188 (1.6)	1/30 (3.3)	2/158 (1.3)	0.408
**5**	**C*03:04**	6/188 (3.7)	1/30 (3.3)	6/158 (3.8)	1.000
**6**	**C*04:01**	20/188 (10.6)	3/30 (10.0)	17/158 (10.8)	1.000
**7**	**C*05:01**	18/188 (9.6)	2/30 (6.7)	16/158 (10.1)	0.743
**8**	**C*06:02**	18/188 (9.6)	1/30 (3.3)	17/158 (10.8)	0.315
**9**	**C*07:01**	30/188 (16.0)	6/30 (20.0)	24/158 (15.2)	0.586
**10**	**C*07:02**	12/188 (6.4)	1/30 (3.3)	11/158 (7.0)	0.694
**11**	**C*07:04**	3/188 (1.6)	1/30 (3.3)	2/158 (1.3)	0.408
**20**	**C*08:01**	1/188 (0.5)	0/30 (0.0)	1/158 (0.6)	1.000
**12**	**C*08:02**	10/188 (5.3)	2/30 (6.7)	8/158 (5.1)	0.662
**13**	**C*12:02**	5/186 (2.7)	0/28 (0.0)	5/158 (3.2)	1.000
**14**	**C*12:03**	18/188 (9.6)	5/30 (16.7)	13/158 (8.2)	0.173
**15**	**C*14:02**	1/187 (0.5)	0/29 (0.0)	1/158 (0.6)	1.000
**16**	**C*15:02**	6/188 (3.2)	1/30 (3.3)	5/158 (3.2)	1.000
**17**	**C*16:01**	6/188 (3.2)	0/30 (0.0)	6/158 (3.8)	0.592
**18**	**C*16:02**	1/188 (0.5)	1/30 (3.3)	0/158 (0.0)	0.160
**19**	**C*17:01**	5/188 (2.7)	1/30 (3.3)	4/158 (2.5)	0.585

HLA-DPA1

		Total (n=190)	Died (n=30)	Survived (n=160)	P
1	**DPA1*01:03**	147/188 (78.2)	25/30 (83.3)	122/158 (77.2)	0.630
2	**DPA1*01:05**	1/188 (0.5)	0/30 (0.0)	1/158 (0.6)	1.000
3	**DPA1*02:01**	36/188 (19.1)	3/30 (10.0)	33/158 (20.9)	0.210
4	**DPA1*02:02**	3/188 (1.6)	2/30 (6.7)	1/158 (0.6)	0.067
5	**DPA1*03:01**	1/188 (0.5)	0/30 (0.0)	1/158 (0.6)	1.000

HLADPB1

		Total (n=190)	Died (n=30)	Survived (n=160)	P
**1**	**DPB1*01:01**	11/173 (6.9)	1/30 (3.3)	10/143 (7.0)	0.692
**2**	**DPB1*02:01**	26/173 (15.0)	7/30 (23.3)	19/143 (13.3)	0.167
**3**	**DPB1*02:02**	2/173 (1.2)	0/30 (0.0)	2/143 (1.4)	1.000
**4**	**DPB1*03:01**	10/173 (5.8)	0/30 (0.0)	10/143 (7.0)	0.214
**5**	**DQB1*03:02**	1/169 (0.6)	0/30 (0.0)	1/139 (0.7)	1.000
**6**	**DPB1*04:01**	58/173 (33.5)	9/30 (30.0)	49/143 (34.3)	0.832
**7**	**DPB1*04:02**	29/173 (16.7)	5/30 (16.7)	24/143 (16.8)	1.000
**8**	**DPB1*05:01**	3/173 (1.7)	1/30 (3.3)	2/143 (1.4)	0.437
**9**	**DPB1*09:01**	1/173 (0.6)	0/30 (0.0)	1/143 (0.7)	1.000
**10**	**DPB1*10:01**	3/173 (1.7)	1/30 (3.3)	2/143 (1.4)	0.437
**11**	**DPB1*11:01**	11/173 (6.9)	2/30 (6.7)	10/143 (7.0)	1.000
**12**	**DPB1*13:01**	5/173 (2.9)	2/30 (6.7)	3/143 (2.1)	0.208
**13**	**DPB1*14:01**	4/173 (2.3)	1/30 (3.3)	3/143 (2.1)	0.537
**14**	**DPB1*15:01**	1/173 (0.6)	0/30 (0.0)	1/143 (0.7)	1.000
**15**	**DPB1*17:01**	4/173 (2.3	0/30 (0.0)	5/143 (3.5)	0.589
**16**	**DPB1*19:01**	2/173 (1.2)	1/30 (3.3)	1/143 (0.7)	0.318

HLA-DQA1

		Total (n=190)	Died (n=30)	Survived (n=160)	P
1	**DQA1*01:01**	30/182 (16.5)	5/27 (18.5)	25/155 (16.1)	0.780
2	**DQA1*01:02**	35/182 (19.2)	3/27 (11.1)	32/155 (20.6)	0.301
3	**DQA1*01:03**	15/182 (8.2)	2/27 (7.4)	13/155 (8.4)	1.000
4	**DQA1*02:01**	28/182 (15.4)	3/27 (11.1)	25/155 (16.1)	0.772
5	**DQA1*03:01**	27/182 (14.8)	7/27 (25.9)	20/155 (12.9)	0.137
6	**DQA1*04:01**	6/182 (3.3)	0/27 (0.0)	6/155 (3.9)	0.594
7	**DQA1*05:01**	41/182 (22.5)	7/27 (25.9)	34/155 (21.9)	0.624

HLA-DQB1

		Total (n=190)	Died (n=30)	Survived (n=160)	P
**1**	**DQB1*02:01**	34/175 (19.4)	6/28 (21.4)	28/147 (19.0)	0.796
**2**	**DQB1*02:02**	3/175 (1.7)	0/28 (0.0)	3/147 (2.0)	1.000
**3**	**DQB1*03:01**	24/175 (13.7)	5/28 (17.9)	19/147 (12.9)	0.548
**4**	**DQB1*03:02**	18/175 (10.3)	3/28 (10.7)	15/147 (10.2)	1.000
**5**	**DQB1*03:03**	6/175 (3.4)	1/28 (3.6)	5/147 (3.4)	1.000
**6**	**DQB1*04:02**	9/175 (5.1)	0/28 (0.0)	9/147 (6.1)	0.358
**7**	**DQB1*05:01**	28/175 (16.0)	3/28 (10.7)	25/147 (17.0)	0.576
**8**	**DQB1*05:02**	5/175 (2.9)	0/28 (0.0)	4/147 (2.7)	1.000
**9**	**DQB1*05:03**	9/175 (5.1)	2/28 (7.1)	7/147 (4.8)	0.638
**10**	**DQB1*06:01**	4/175 (2.3)	0/28 (0.0)	4/147 (2.7)	1.000
**11**	**DQB1*06:02**	17/175 (9.7)	4/28 (14.3)	13/147 (8.8)	0.482
**12**	**DQB1*06:03**	9/175 (5.1)	2/28 (7.1)	7/147 (4.8)	0.638
**13**	**DQB1*06:04**	8/175 (4.6)	2/28 (7.1)	6/147 (4.1)	0.616
**14**	**DQB1*06:09**	1/175 (0.6)	0/28 (0.0)	1/147 (0.7)	1.000

HLA-DRB1

		Total (n=190)	Died (n=30)	Survived (n=160)	P
**1**	**DRB1*01:01**	6/160 (3.8)	3/23 (13.0)	3/137 (2.2)	**0.039**
**2**	**DRB1*01:02**	6/160 (3.8)	2/23 (8.7)	4/137 (2.9)	0.207
**3**	**DRB1*01:03**	2/160 (1.3)	0/23 (0.0)	2/137 (1.5)	1.000
**4**	**DRB1*03:01**	28/160 (17.5)	4/23 (17.4)	24/137 (17.5)	1.000
**5**	**DRB1*03:02**	1/160 (0.6)	0/23 (0.0)	1/137 (0.7)	1.000
**6**	**DRB1*04:01**	1/159 (0.6)	0/23 (0.0)	1/136 (0.7)	1.000
**7**	**DRB1*04:02**	2/160 (1.3)	1/23 (4.3)	1/137 (0.7)	0.268
**8**	**DRB1*04:04**	2/160 (1.3)	1/23 (4.3)	1/137 (0.7)	0.268
**9**	**DRB1*04:05**	1/160 (0.6)	0/23 (0.0)	1/137 (0.7)	1.000
**10**	**DRB1*04:07**	0/159 (0.0)	0/23 (0.0)	0/136 (0.0)	1.000
**11**	**DRB1*04:10**	1/159 (0.6)	0/23 (0.0)	1/136 (0.7)	1.000
**12**	**DRB1*07:01**	32/160 (20.0)	4/23 (17.4)	28/137 (20.4)	1.000
**13**	**DRB1*08:01**	2/160 (1.3)	0/23 (0.0)	2/137 (1.5)	1.000
**14**	**DRB1*08:02**	1/159 (0.6)	0/23 (0.0)	1/136 (0.7)	1.000
**15**	**DRB1*08:04**	1/159 (0.6)	0/23 (0.0)	1/136 (0.7)	1.000
**16**	**DRB1*09:01**	2/160 (1.3)	0/23 (0.0)	2/137 (1.5)	1.000
**17**	**DRB1*10:01**	5/160 (3.1)	0/23 (0.0)	5/137 (3.6)	1.000
**18**	**DRB1*11:01**	3/160 (1.9)	0/23 (0.0)	3/137 (2.2)	1.000
**19**	**DRB1*11:02**	2/160 (1.3)	0/23 (0.0)	2/137 (1.5)	1.000
**20**	**DRB1*11:04**	4/160 (2.5)	0/23 (0.0)	4/137 (2.9)	1.000
**21**	**DRB1*11:06**	1/160 (0.6)	1/23 (4.3)	0/137 (0.0)	0.144
**22**	**DRB1*12:01**	1/159 (0.6)	0/23 (0.0)	1/136 (0.7)	1.000
**23**	**DRB1*13:01**	15/160 (9.4)	2/23 (8.7)	13/137 (9.5)	1.000
**24**	**DRB1*13:02**	3/160 (1.9)	1/23 (4.3)	2/137 (1.5)	0.374
**25**	**DRB1*14:01**	2/160 (1.3)	0/23 (0.0)	2/137 (1.5)	1.000
**26**	**DRB1*14:02**	4/160 (2.5)	0/23 (0.0)	4/137 (2.9)	1.000
**27**	**DRB1*14:04**	4/159 (2.5)	1/23 (4.3)	3/136 (2.2)	0.468
**28**	**DRB1*15:01**	13/160 (8.1)	4/23 (17.4)	9/137 (6.6)	0.096
**29**	**DRB1*15:02**	4/160 (2.5)	0/23 (0.0)	4/137 (2.9)	1.000

**30**	**DRB1*15:03**	1/159 (0.6)	0/23 (0.0)	1/136 (0.7)	1.000
**31**	**DRB1*16:01**	6/159 (3.8)	0/23 (0.0)	6/136 (4.4)	0.594
**32**	**DRB1*16:02**	1/159 (0.6)	0/23 (0.0)	1/136 (0.7)	1.000
**33**	**DRB3*99:01**	1/159 (0.6)	0/23 (0.0)	1/136 (0.7)	1.000

HLA-DRB3

		Total (n=190)	Died (n=30)	Survived (n=160)	P
**1**	**DRB3*01:01**	22/183 (12.0)	5/30 (16.7)	17/153 (11.1)	0.368
**2**	**DRB3*02:02**	43/183 (23.5)	7/30 (23.3)	36/153 (23.5)	1.000
**3**	**DRB3*03:01**	8/183 (4.4)	3/30 (10.0)	5/153 (3.3)	0.125
**4**	**DRB3*99:01**	110/183 (60.1)	15/30 (50.0)	95/153 (62.1)	0.228

HLA-DRB4

		Total (n=190)	Died (n=30)	Survived (n=160)	P
**1**	**DRB4*01:01**	5/183 (2.7)	2/30 (6.7)	3/153 (2.0)	0.189
**2**	**DRB4*01:03**	20/183 (10.9)	0/30 (0.0)	20/153 (13.1)	**0.048**
**3**	**DRB4*99:01**	158/183 (86.7)	28/30 (93.3)	130/153 (85.0)	0.377

HLA-DRB5

		Total (n=190)	Died (n=30)	Survived (n=160)	P
**1**	**DRB5*01:01**	19/188 (10.1)	3/30 (10.0)	16/158 (10.1)	1.000
**2**	**DRB5*01:02**	3/188 (1.6)	0/30 (0.0)	3/158 (1.9)	1.000
**3**	**DRB5*99:01**	166/188 (88.3)	27/30 (90.0)	139/158 (88.0)	1.000
